# Newborn care and knowledge translation - perceptions among primary healthcare staff in northern Vietnam

**DOI:** 10.1186/1748-5908-6-29

**Published:** 2011-03-29

**Authors:** Leif Eriksson, Nguyen Thu Nga, Dinh P Hoa, Lars-Åke Persson, Uwe Ewald, Lars Wallin

**Affiliations:** 1 International Maternal and Child Health (IMCH), Department of Women's and Children's Health, Uppsala University, Uppsala, Sweden; 2 Vietnam Sweden Uong Bi General Hospital, Quang Ninh, Vietnam; 3 Hanoi School of Public Health, Hanoi, Vietnam; 4 Neonatology, Department of Women's and Children's Health, Uppsala University, Uppsala, Sweden; 5 Department of Neurobiology, Care Sciences and Society, Division of Nursing, Karolinska Institutet and Clinical Research Utilization (CRU), Karolinska University Hospital, Stockholm, Sweden

## Abstract

**Background:**

Nearly four million neonatal deaths occur annually in the world despite existing evidence-based knowledge with the potential to prevent many of these deaths. Effective knowledge translation (KT) could help to bridge this know-do gap in global health. The aim of this study was to explore aspects of KT at the primary healthcare level in a northern province in Vietnam.

**Methods:**

Six focus-group discussions were conducted with primary healthcare staff members who provided neonatal care in districts that represented three types of geographical areas existing in the province (urban, rural, and mountainous). Recordings were transcribed verbatim, translated into English, and analyzed using content analysis.

**Results:**

We identified three main categories of importance for KT. Healthcare staff used several channels for acquisition and management of knowledge (1), but none appeared to work well. Participants preferred formal training to reading guideline documents, and they expressed interest in interacting with colleagues at higher levels, which rarely happened. In some geographical areas, traditional medicine (2) seemed to compete with evidence-based practices, whereas in other areas it was a complement. Lack of resources, low frequency of deliveries and, poorly paid staff were observed barriers to keeping skills at an adequate level in the healthcare context (3).

**Conclusions:**

This study indicates that primary healthcare staff work in a context that to some extent enables them to translate knowledge into practice. However, the established and structured healthcare system in Vietnam does constitute a base where such processes could be expected to work more effectively. To accelerate the development, thorough considerations over the current situation and carefully targeted actions are required.

## Background

Despite the existence of cost-effective, evidence-based practices, nearly four million neonatal deaths occur and more than three million babies are stillborn each year [1,2]. Recent estimations indicate that > 70% of all neonatal deaths could be averted by universal coverage of evidence-based interventions (*e.g*., skilled attendance at birth, exclusive breastfeeding, and hypothermia management) [1]. Successful implementation of such interventions in low- and middle-income countries, in which almost all (99%) neonatal deaths take place, would have a strong impact on neonatal health and survival. Therefore, investments in translating evidence into practice should be a global undertaking of high priority [3,4]. Knowledge translation (KT) is a field in healthcare science and practice that aims to improve health and quality of healthcare through "a dynamic and iterative process that includes synthesis, dissemination, exchange and ethically sound application of knowledge" [5]. The World Health Organization (WHO) has placed KT high on its agenda and claims that bridging the gap between what is known and what is done is one of the most important future challenges [6,7]. However, globally there is still a lack of knowledge on the effectiveness of different implementation strategies [8-10]. One aspect of this scarcity is that KT is mainly investigated in rich countries [11-13], and among the KT studies conducted in low- and middle-income countries, many are poorly performed, which further limits the opportunity to draw valid conclusions [10].

In Quang Ninh province, which is located in the northeastern part of Vietnam, the neonatal mortality rate (NMR) was 16 deaths per 1,000 live births in 2005 [14]. The NMR in the districts in the Quang Ninh province ranged from 10 to 44 per 1,000, with the highest proportions of home deliveries occurring in the high mortality districts [15]. This situation contributed to the rationale for implementing the study Neonatal Health-Knowledge into Practice (NeoKIP, trial registration ISRCTN44599712), in which the effectiveness of a KT intervention for improved neonatal health and survival is investigated. In the NeoKIP study, we use the Promoting Action on Research Implementation in Health Services (PARIHS) framework [16] to theoretically frame the study. The PARIHS framework highlights the importance of three cornerstones for successful change of clinical practice: evidence, context, and facilitation. Knowing that the available evidence for newborn healthcare is strong, NeoKIP focuses on assessing the effectiveness of facilitation in a Vietnamese context. The PARIHS framework suggests that the evidence available for change of clinical practice can be derived from four types of knowledge base: research, clinical experiences, patient views, and the local context [17]. Furthermore, contextual factors in the form of culture, leadership, evaluation, and resources are important to consider when translating evidence into practice [18,19]. In Vietnam, the Ministry of Health launched practice guidelines for reproductive healthcare (here called the National Guidelines) [20] in 2003 in an effort to increase staff use of evidence-based recommendations and thus improve the healthcare for pregnant women and neonates. However, our research group reported from the NeoKIP baseline survey in 2006 that primary healthcare staff had scarce knowledge on evidence-based practices in neonatal health and rarely used the National Guidelines [21]. Further, Vietnam is one of few countries that has integrated traditional medicine (TM) into the healthcare system [22]. Some traditional practices are therefore recommended by and used within public healthcare [23] and might compete with evidence-based practices (*e.g*., those recommended by the National Guidelines). TM is commonly used by all ethnic groups in Vietnam but more frequently by ethnic minority groups [24]. The Vietnamese context provides rich opportunities to study aspects of KT in a middle-income country.

Before implementing the facilitation intervention, a qualitative study was performed within the NeoKIP project, with the aim to explore how knowledge was translated into practice among primary healthcare staff involved in the care of pregnant women and neonates in Quang Ninh province, Vietnam. Specifically, we wanted to investigate how healthcare personnel acquired new knowledge, how change of clinical practice was accomplished, and how the use of TM interacted with evidence-based practices.

## Method

### Setting

Quang Ninh province is located in northeastern Vietnam along the coast bordering China. The province has approximately one million inhabitants, and 35% are considered living under poor conditions [25]. Kinh is the largest ethnic group in Quang Ninh, comprising a proportion of the population comparable to that of the entire country's (~85%). The remaining population in Quang Ninh can be divided into 20 ethnic minority groups. These groups differ in language and culture between each other and when compared with the ethnic majority group Kinh. The province is administratively divided into 14 districts and 184 communities. Urbanization and economic development are rapid in Vietnam, but still a large proportion of the population in Quang Ninh lives in rural or mountainous areas. The province, however, is considered rich in comparison with other Vietnamese provinces [24]. Coal mining and tourism are major sources of income in Quang Ninh. The healthcare system in the province consists of 1 regional hospital, 1 provincial hospital, 16 district hospitals, and 187 community health centres (CHCs). Medical doctors, assistant doctors, midwives, and nurses constitute the staff working at the CHCs. Medical doctors in Vietnam are trained for six years at a medical college, while assistant doctors, midwives, and nurses are trained for two or three years at a nursing school. In each CHC, there are three to six staff members working, whereof one or two, primarily midwives and assistant doctors, are responsible for reproductive healthcare. One of the CHC staff members is also responsible for TM. Each village has its own village health worker (VHW) who has basic healthcare training and is employed part time by the CHC.

### Study sample and data collection

We used a purposive sampling strategy [26] to include CHC staff working with neonatal care in three districts that represented the types of geographical areas existing in the province (mountainous, rural, and urban). A geographical representative sample of CHCs from each of the three districts was selected for this study, and staff members working with neonatal care from the selected CHCs were invited to share their views. This arrangement resulted in six groups with seven to eight individuals coming from different communities in each group. Three groups were planned to exclusively include assistant doctors and medical doctors and the other three groups to include midwives and nurses; however, the groups did not become completely homogeneous (Table 1). A majority of the participants from the mountainous district were from the ethnic minority group Dao, whereas in the other types of district almost all the participants were Kinh.

**Table 1:** Group composition and characteristics of the focus groups

Group	District type	Age (range in years)	Sex (female/male)	Ethnic group (Kinh/Dao/Sin Dui)	Profession (medical doctor/assistant doctor/midwife/nurse)
**1**	Rural	39-46	5/3	8/0/0	2/6/0/0

**2**	Rural	25-45	7/0	7/0/0	0/3/2/2

**3**	Mountainous	36-48	4/3	2/5/0	2/5/0/0

**4**	Mountainous	27-44	7/0	3/4/0	0/1/6/0

**5**	Urban	37-51	8/0	7/0/1	2/5/1/0

**6**	Urban	24-46	7/0	7/0/0	1/2/3/1

Focus-group discussion (FGD) was used as the method of data collection. The FGDs were conducted in Vietnamese and led by a moderator (a physician from Vietnam and the second author [NTN] of this paper with previous experience of moderating FGDs). A note-taker (who was a trained data collector within the NeoKIP project) and an observer (a Swedish registered nurse and first author of this paper) kept track of nonverbal activities during the group discussions. An interview guide with six open-ended questions was used (Additional File 1). Some probing questions were used to help the moderator with less talkative groups. The interview questions and probes, generated through discussions in the NeoKIP research group, were based on issues identified during the baseline assessment that were considered in need of clarification before the start of the facilitation intervention. The FGDs lasted from 90 to 120 minutes, including a short break. All FGDs were recorded with a portable minidisc recorder. The moderator, note-taker, and observer met after each FGD to discuss the content and lessons learnt for the next FGD.

### Data analysis

The audio-recorded material from the FGDs was transcribed verbatim, material from the note-taker was added, and an idiomatic translation was conducted of all the material from Vietnamese into English. The translations were checked by the two Vietnamese authors (NTN and DPH) of this paper. Manifest qualitative content analysis was used to analyze the English transcriptions [27]. The first step in the analysis was to read the material several times, then identify meaning units, condense the meaning units, and label them with codes. Thereafter, an abstraction process took place by which the codes were sorted into subcategories, the subcategories were sorted into categories, and finally, the categories were sorted into main categories [28]. An example of the abstraction process is presented in Table 2. The analytic process included a close collaboration between the first (LE) and the last (LW) authors, and all discrepancies in the analysis were discussed until consensus was reached.

**Table 2:** Example of the abstraction process

Meaning unit	I think that when there is a new guideline or a treatment protocol, we all should assemble at one place (e.g., at hospital or somewhere else) in order to have a short training session so that we can learn effectively and build on our successes. Furthermore, there should be refresher training or review training every year.
**Condensed meaning unit**	When having a new guideline, we should all gather at hospital for a short training session and have refresher training once a year.

**Codes**	When having new guidelines, all should gather and train. Refresh training on guidelines once a year.

**Subcategory**	New guidelines should require training of staff.

**Category**	Training

**Main category**	Knowledge acquisition and management

### Ethical considerations

The study was approved by the Ministry of Health in Vietnam, the Provincial Health Bureau in Quang Ninh, and the Research Ethics Committee at Uppsala University, Sweden. Participation in a FGD was voluntary. The data could not be identified and were handled with confidentiality.

## Results

**Figure 1:**
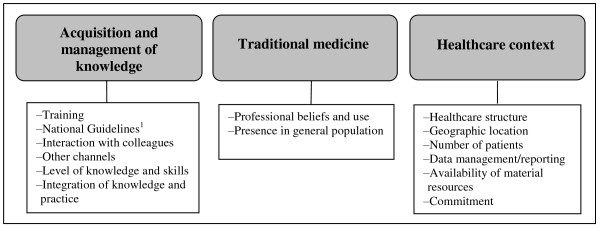
**The three main categories and categories derived from the analysis**. ^1^National standards and guidelines for reproductive health care services (2003) by the Ministry of Health in Vietnam

The analysis of data resulted in three main categories (Figure 1) summarizing primary healthcare staff views from the six FGDs: (1) acquisition and management of knowledge, (2) traditional medicine, and (3) issues related to the healthcare context. The results are presented under these three main categories (see Additional File 2 for all levels of categories).

### Acquisition and management of knowledge

This main category reflects the FGD participants' many views on how health knowledge was acquired and managed. Training was perceived as important as well as the best way to acquire knowledge. Training included both theoretical and practical training that aimed at improving staff knowledge and skills in their present position at the primary healthcare level. Several of the participants requested additional training in different areas (*e.g*., in obstetrics and paediatrics). Some dissonance was noted regarding the place for training (at hospitals or at CHCs) and the required length of the training for best results. There was a common opinion that all staff members needed training, not only for a select few. The care workers at the CHCs reported that their work schedule was arranged on a rotating basis. Such varied shift rotation meant that the care workers worked various shifts, including day, evening, night, weekday, and weekend shifts, implying that the staff members met patients with a variety of problems. This situation motivated staff for training in different fields (regardless of their profession and specialization) in order to be able to provide a mixture of services to people seeking care at the CHCs.

The content of the National Guidelines was considered relevant, but this tool was rarely used. The availability of the National Guidelines and any methods employed to disseminate the guidelines differed among communities. Most participants claimed that there had been a poor introduction of the National Guidelines, a problem that was seen as common in other similar situations.

I think that when there is a new guideline or a treatment protocol, we all should assemble at one place (*e.g*., at hospital or somewhere else) in order to have a short training session so that we can learn effectively and build on our successes. Furthermore, there should be refresher training or review training every year. (Doctor, mountainous group)

Interaction with colleagues was experienced as a common way of knowledge acquisition. However, CHC staff mainly consulted colleagues at the primary healthcare level, and contact with staff at higher levels of the healthcare system was rarely taken.

Health facilities should collaborate with each other. It would be practical and useful if the district hospital staff could visit the CHC once a week to supervise our daily work and then provide support in a timely manner. (Doctor, rural group)

Other channels to acquire knowledge were, for example, textbooks, documents from different gatherings (retraining occasions and workshops), and information provided by pharmaceutical drug companies. However, there was no consistency in the availability of these sources of knowledge at the primary healthcare level. The study participants considered it difficult to determine which information among the several sources to use in their daily work. Mass media was also a channel of knowledge; in particular, the Ministry of Health's newspaper ("Health and Life Newspaper") was considered important [29]. Computers with internet connections were not available as a means to acquire knowledge at the CHCs: 'We never touch the computer keys'. (Assistant doctor, rural group)

Study participants emphasised that extensive knowledge and well-developed skills were important in providing high-quality care. However, they also expressed that the current level of staff knowledge and skills was often poor at the CHCs, which resulted in negative consequences for patients and a weakening of the healthcare system.

There are rough hands [staff with inadequate knowledge and skills] working with obstetrics and paediatrics at the CHC; sometimes patients get scared when they see those hands. We need to select hands that can provide gentle service and for the health of the women and children; hands should be small and not rough. (Assistant doctor, rural group)

We also noted that the ability to integrate knowledge and practice was an individual factor that varied between staff members. Some contributions in the FGDs revealed that participants had integrated evidence-based knowledge into practices, whereas others indicated that either the knowledge or the practical-implementation component was missing. Participants expressed that they lacked knowledge on new practices and treatment regimens, despite their being recommended in guidelines and already established as routines at hospitals.

What I was taught in theory and what I observed during clinical practice at the regional hospital is different. At the regional hospital, we were told to absolutely not hold the baby upside down but place the baby on the mother's belly after the delivery. So, I applied the practice from the regional hospital in our health station with three cases, but I don't know why to do that. (Assistant doctor, mountainous group)

### Traditional medicine

In this paper the concept of TM, derived from WHO, has a broad meaning and refers to customs and treatments that use medication as well as nonmedication therapies [22], without differentiating between practices used within and outside the healthcare system. The FGDs revealed that TM had a prominent position in terms of knowledge that both the healthcare staff and the general population considered useful in the care of pregnant and postpartum women and their newborns. The study participants were eager to share their experiences and perceptions of TM in this field. Different TM practices were described regarding women's abdominal pain, contraction of the uterus, haemorrhage, hygiene, milk production, and nutrition. TM was mainly applied among neonates for symptoms such as cough, fever, hygiene, jaundice, pain, rash, skin infection, and thrush. FGD participants had knowledge of various customs and practices (*e.g*., postpartum bathing of the mother and the newborn child with specific herbs, leaves, or roots that were described as beneficial) commonly used in society and recommended to patients by CHC staff.

...it is unlikely that the neonate will get a cold when they are bathed with traditional medicine. (Assistant doctor, mountainous group)

When we see a baby with jaundice, we just tell the parents to bathe the baby with Cockscomb broth and we do not ask them to have any laboratory tests taken. (Doctor, mountainous group)

Some participants in the FGDs reported that, at times, they preferred to use TM instead of evidence-based medicine, whereas others stated that it could be a conflict for them to decide when to use what. Examples were also given underlining that staff were opposed to certain TM practices but tolerant of them because the general population used such treatments.

It has not been scientifically tested, but when the baby cries, the family should burn the Mugwort because the smoke stops the baby from crying. So I think that the smoke of Mugwort helps to clear the baby's nose. I am personally against this practice, but I think it is alright that they use it. (Assistant doctor, rural group)

According to the study participants, TM was used to a greater extent in the mountainous and rural communities and in areas with a higher proportion of ethnic minority groups. However, TM was also most often the first choice of treatment of mild conditions for many primary healthcare personnel.

In my CHC we have some herbal trees [*i.e*., trees growing in the garden of the CHCs from which the leaves are used] in order to introduce the simplest traditional methods for women and children with common diseases. If the herbal medicines are unsuitable, we will switch to western medicine, which is a higher level of treatment. (Assistant doctor, urban group)

### Healthcare context

Many factors of importance for KT were linked to the healthcare context. For example, there were few patients seeking care at some CHCs because many community members bypassed the primary care level and instead directly consulted the hospitals. Participants questioned whether it was possible to be skillful with such a low level of workload as described for some of the CHCs.

If there are no deliveries, or once in a while we assist a delivery, or there are only two to three deliveries per year, we may forget what we have learned. (Midwife, mountainous group)

Further, data management and data reporting at the community healthcare level were considered important but not functioning well. The availability of material resources (equipment and drugs) was also insufficient. However, some resources were available but not used because the staff had not received any training in their use.

We received an electric suction machine without having anyone to teach us how to use it and there was no user manual in the box either. We learnt how to use the machine when we saw people use it at the hospital, so we imitated. In fact, I bet there may be many other CHCs where they don't know how to use their equipment. (Midwife, rural group)

The geographic location of a CHC was considered an important issue. Staff from mountainous and rural CHCs expressed that they had more limitations than did staff at urban CHCs. For example, personnel from mountainous and rural CHCs claimed that they had less qualified staff, lack of training, and fewer material resources in comparison with more urban CHCs. They also reported difficulties in referring patients to hospitals because of the long travel distances.

Study participants pointed out that acquiring and managing knowledge is a process that takes time, needs good support, and is dependent on the capacity and commitment of the individual staff. For example, the VHWs were described as important persons who work closely with families in the community but that they receive low pay and are often not committed to their work. This lack of commitment was regarded as a strong contributor to the perceived poor quality of services provided by some VHWs. Support from higher levels in the healthcare system was considered necessary in order to implement change in clinical practice at the community level. However, such support was usually not available. The hierarchical structure of the health system in the province seemed to impede knowledge dissemination and uptake. There was a lack of interaction between healthcare levels, and there was mostly a one-way flow of information (from the top to the bottom). The participants in the FGDs experienced that, instead of giving appreciation and guidance, staff from higher levels of the health system often criticized the work at the CHC.

When referring a patient to a hospital, the parents often hear from the doctors at the hospital: If you had been 10 or 15 more minutes later, the child would have died. The parents will then blame us for what they think are improper examination and diagnosis. This is a disaster at our level and it creates difficulties. (Assistant doctor, rural group)

## Discussion

This study explored the views of primary healthcare staff on issues related to the KT processes at their workplaces. The analysis of the FGDs resulted in three main categories: the acquisition and management of knowledge, TM, and factors related to the healthcare context. In the following discussion we will elaborate on specific findings within these main categories, where the current situation seems to impede basic processes of KT, but if changed, could instead facilitate beneficial development. The PARIHS framework will be used to discuss and summarize the major findings.

The different channels for knowledge acquisition were central to this study, which links well with the diffusion of innovation theory, a theory suggesting that innovation is communicated over certain channels [30]. The National Guidelines were one of the channels for communication of new knowledge. However, the low use of the National Guidelines previously reported [21] was confirmed by statements in the focus groups in the present study. Participants claimed that the infrequent use of the guidelines was because of their poor introduction in 2003. Primary healthcare staff also referred to other guiding policy documents available at the health centres. This range of recommendations seemed to confuse the staff in their choice of what to rely on for specific care situations. Today, the internet is a highly used electronic medium for communication and for the exchange of knowledge. However, in this study region there was no internet access at the CHCs, which further underlines the importance of having clear guidance when implementing recommendations to ensure that all members of the primary healthcare staff know how to use them for best practice in their work.

Training was perceived to be the most important means of acquiring knowledge. According to Grol and Grimshaw [31], education can be an effective way of changing practitioners' behaviour, particularly if it involves elements of interaction and discussion in small groups. In fact, the participants in the FGDs claimed that staff at the CHCs were interacting and exchanging knowledge to some extent. However, the participants asked for more interaction with staff at different healthcare levels, an interaction mode that seemed to be lacking. Laverack and Tuan [32] verify that communication across healthcare levels rarely occurs in Vietnam: the flow of information mainly goes from higher to lower levels as opposed to a two-way interaction between levels. We also identified that more didactic and formal top-to-bottom approaches of information dissemination and education were common and that the staff approved of these approaches. This appreciation of the traditional didactic education style is questionable, however. We believe that to be effective, education should have ingredients of interaction (*e.g*., through small group discussions and audit and feedback) [4,31,33]. A recent study in northern Vietnam, in which researchers used participatory methods when introducing an educational programme for community health leaders, demonstrated promising results in learning capacity, and the health leaders expressed enthusiasm for this mode of gaining knowledge [34]. Further, Rycroft-Malone [35] suggests that a healthcare context that decentralizes decisions, that puts emphasis on the relationship between managers and workers, and that uses a management style that is facilitative rather than directive will create a learning organization (*i.e*., an organization that considers individuals, group processes, and organizational systems). An introduction of more participatory approaches in the study province could increase the communication between healthcare levels, which the study participants requested, and thus enhance the process of uptake and management of knowledge.

TM in Vietnam derives from Chinese medicine and indigenous practices from Vietnamese ethnic minority groups [36]. The Vietnamese form of TM has influenced both the lifestyle of the population and the care provided within the healthcare system [22,23]. When participants revealed their views of TM in relation to KT, the statements mainly consisted of descriptions of the use of TM by the general population, but some examples were also included from their professional life. The findings suggest that TM has a strong position in Vietnam, especially among ethnic minority groups [24]. The TM norms can function as a barrier to change [30], explaining why a far 'newer' concept of evidence-based practice, such as the recommendation of delaying bathing of newborns (to avoid hypothermia) [37], has met with difficulties in being accepted and implemented in some areas. Clashes between cultures within organizations often lead to suboptimal conditions for providing quality care [18], which may explain why staff, having two cultures (evidence based and traditional) to rely upon, had difficulties in determining what kind of practice they should consider. In contrast, at other CHCs, evidence-based medicine and traditional methods appeared to function well together without competition or conflict. Vietnam has decided to emphasize the development of TM and to promote a rational use of both modern and traditional therapies [38]. Such decisions are consistent with WHO's policy regarding the importance of gathering scientific evidence for different TM practices [22]. The rationale for this is obvious, exemplified by the discovery of the antimalarial drug artemisinin in China, which has been vitally important in malaria treatment, replacing previous drugs against which the parasite had developed resistance [39]. Although Vietnam has regulations for the use of TM in healthcare [23], many of the practices in the general population are not based on official recommendations. Moreover, there are many active TM practitioners without formal education in the field [36]. However, even if staff members at CHCs sometimes disagree with the traditional methods used outside the healthcare sector, they are not actively opposing this use. Study participants also revealed that the CHC staff advised their patients in the perinatal period to use TM despite the absence of any such recommendations in the National Guidelines. To strengthen and support regulations of the use of TM and to make the KT process more straightforward, healthcare staff may need to reflect more critically on how they use and advise clients to use TM.

The low level of activity at some CHCs could lead to difficulties in maintaining an adequate level of knowledge. This concern is highly relevant. Recently, we have shown that healthcare facilities in Quang Ninh province with few deliveries have a higher neonatal mortality rate [40], indicating that such facilities have difficulties maintaining a high standard of care in delivery [41]. The local population is most likely aware of such limitations and thus prefer to seek care at higher levels, even if such institutions are located far away. This, however, will not be an option for poorer segments of the population. In addition to a low level of activity, many CHCs lack essential equipment for care in delivery [21], which might result in further difficulties in how to use and sustain knowledge. It was also described that CHCs might possess certain equipment (*e.g*., the electric suction machine) but lack knowledge on how and when to use such equipment. In the case of the suction machine, the lack of knowledge did not prevent CHC staff from using it. That particular CHC appeared, as Rogers [30] describes it, to have had an early adopter who speeded up the adoption process by imitating hospital staff. This was an innovative and common process according to the study participants. However, there are risks linked to this behaviour. Routine airway suction of newborn infants is not supported by current evidence [42].

Motivation among healthcare staff is described as an important factor for increased quality of care [43]. In a qualitative study among rural health workers in North Vietnam, Dieleman and colleagues [44] identified a number of factors that affected staff motivation (*e.g*., appreciation, training, respect, and a stable work situation) and demotivation (*e.g*., low income, difficult transportation, and lack of information and training). These findings may help to explain why VHWs, who are poorly educated and low paid, were perceived as uncommitted by CHC staff in our study. For example, to get a sufficient income every month, the VHWs are forced to have additional jobs, which influences focus and quality of work as a VHW. Solving such problems could potentially enhance performance and function, not only of the VHWs and CHCs but also of the entire healthcare system.

## Limitations

In this study we wanted to capture experiences, perceptions, and norms of primary healthcare personnel. Focus groups, which generate information from a host of people through interactions [26], were therefore chosen as the method for data collection. In general, the participants expressed interest and actively contributed to the FGDs. A member of the research team, a Vietnamese paediatrician, moderated the FGDs. Because of her knowledge on neonatal care and previous experience in leading focus groups, we anticipated that she would have good opportunities to stimulate interaction between the participants. However, in Vietnamese culture, criticism is a sensitive issue, especially in the presence of a superior. Thus, a potential limitation of this study might be the fact that the moderator had superior rank in the healthcare system than focus group participants did. We separated medical doctors and assistant doctors from midwives and nurses in the focus groups in order to achieve a climate in the discussion that would allow everyday practices to be freely discussed. This strategy proved successful in the groups with doctors, but the groups of midwives and nurses that had at least one doctor in the group were less talkative, suggesting that the doctor and/or the moderator unintentionally may have hampered communication. Despite the fact that several problems of the healthcare system were brought up in the discussions, we cannot disregard the possibility that the profession of the moderator affected exchange of experiences and perceptions.

The study participants came from the three types of setting that exist in the study province (urban, rural, and mountainous); therefore, the findings might be indicative of other districts in the study province. However, many CHCs in Vietnam do not have staff representing all the ethnic groups living in their communities [24]. This drawback was also the case in our study sample, which might have had implications for our findings (*i.e*., not voicing the perceptions or representing the reality of the nonrepresented groups). Further, we did not ask the participants to differentiate between TM that the Vietnamese healthcare system accepts and other TM practices used by the public. Such clarification would have been helpful in gaining a deeper understanding into the complexity of TM. However, this limitation first became evident during the analytic process and, therefore, could not be feasibly addressed. Having authors from Sweden and Vietnam conducting the analysis in English carries an inherent risk in terms of losing important information across the translation and analytic processes. However, we suggest that the credibility of the study might actually have been strengthened by the inclusion of authors of cross-national backgrounds through enriched dialogue of the findings. Among the specific aims in this study, we found that investigating how change of clinical practice was accomplished was more difficult to realize than the other aims. One reason for this shortcoming might be that primary healthcare staff work in a healthcare system that does not encourage staff at this level to initiate changes.

## Summary

To summarize the major findings of this study, we need to refer to the evidence (*i.e*., research, clinical experiences, patient views, and local context) and context (*i.e*., culture, leadership, evaluation, and resources) cornerstones of the PARIHS framework. Knowledge from research was available for CHC staff through several knowledge channels. Yet the participants claimed, for different reasons, that the use of these channels was insufficient. Further, some CHCs lacked resources and were systematically bypassed by patients, indicating difficulties in acquiring the needed clinical experience to maintain knowledge and skills. As a way to enhance learning, the participants requested increased interaction between staff at different levels in the healthcare system. We believe this request is important for the beneficial development of staff competence and clinical practice, although the context has not yet been receptive to such change. Reflection over the widespread use of TM appears to be an important but somewhat neglected issue at the primary healthcare level. The VHWs, who were recognized as a key but underused asset in the Vietnamese healthcare system, might be engaged in increasing evaluation processes by establishing better contact with patients, gaining knowledge on patient views, and increasing the knowledge of the local context by obtaining more correct data reported from the village level to the CHC. To enhance the contribution of VHWs, not only are increased resources for higher salaries necessary, but a change in the existing culture is also required. We believe that many of the obstacles identified in our findings could be recognized and averted with a change in leadership style at both central and local levels in the Vietnamese healthcare system.

This study indicates that the primary healthcare staff personnel in the investigated province work in a context that, to some extent, enables them to translate knowledge into practice. However, the established and structured healthcare system in Vietnam constitutes a base where such processes could be expected to work more effectively. To accelerate the development of KT, thorough considerations over the current situation and carefully targeted actions are required.

## Competing interests

The authors declare that they have no competing interests.

## Authors' contributions

LE and LW designed the study, with assistance from NTN, LÅP, and UE. NTN moderated all the focus group discussions and, together with DPH, assured that translations were correct. LE was responsible for data analysis and drafted the manuscript, with assistance from LW. All authors have read and approved the final manuscript.

## Supplementary Material

Additional file 1 **Interview guide**. Interview guide for the focus group discussions with main questions (in bold) and probing questions. Click here for file

Additional file 2 **All levels of categories from the analysis**. A detailed presentation of all main categories, categories, and subcategories derived from the analysis. Click here for file
