# Supplementary material for: Newborn care and knowledge translation - perceptions among primary healthcare staff in northern Vietnam
Source: Implement Sci. 2011 Mar 29;6:29. doi: 10.1186/1748-5908-6-29 (PMC3080332; doi:10.1186/1748-5908-6-29)
Supplement: Additional file 1 — Interview guide. Interview guide for the focus group discussions with main questions (in bold) and probing questions. [file 1748-5908-6-29-S1.DOC]

# Additional file 1: interview guide

1. **All of you have basic medical training and some of you are specialized in some areas. Can you shortly describe how you experienced your medical training/specialization?**
2. **How do you currently keep yourself updated and acquire new knowledge in your professional field?**
3. **In your opinion, what is the best way of acquiring new knowledge?** For example, if you need/want to learn more about a specific area, how do you proceed at your place of work?
4. **The Ministry of Health launched national standards and guidelines for reproductive healthcare services in 2003. Have you received and seen these guidelines? If yes, can you describe the dissemination and use of these guidelines?** Were there any activities when the guidelines were disseminated to your community health centre (CHC)? Do you currently have them at your place of work? Do you use them? Are the guidelines useful? Is it important for you to have this kind of guidance in your daily work?
5. **How do you change care routines/practices at your place of work?** Who makes the decision if or when routines/practices are to change? Is someone sending you an information letter about, for example, new routines? Is someone coming to your CHC to inform you about new routines/practices? Are you going for a course to learn, etc.? Explain how decisions and procedures are enacted when change is taking place at your place of work.
6. **In what way is traditional medicine used in the care of pregnant women and the newborn child in your community?** Do you practice such traditions? Are such procedures always performed or only on special occasions?
