# Supplementary material for: Newborn care and knowledge translation - perceptions among primary healthcare staff in northern Vietnam
Source: Implement Sci. 2011 Mar 29;6:29. doi: 10.1186/1748-5908-6-29 (PMC3080332; doi:10.1186/1748-5908-6-29)
Supplement: Additional file 2 — All levels of categories from the analysis. A detailed presentation of all main categories, categories, and subcategories derived from the analysis. [file 1748-5908-6-29-S2.DOC]

# Additional file 2: all levels of categories from the analysis

| Acquisition and management of knowledge | Traditional medicine (TM) | Healthcare context |
| --- | --- | --- |
| **Training**   - Training is the best method to update knowledge - Training to get staff working according to new methods - New guidelines should require training of staff - Need for more training in obstetrics and paediatrics - Staff in remote areas need training - Need for more training in management of community health centres (CHCs) - Funding is needed for training - Training can be provided at many levels – convenient at hospitals - Long vs. short training - Refresher training course every year - All staff need training   **National Guidelines**^1^   - Availability of National Guidelines at CHC varies - Inconsistency in method of National Guidelines dissemination - Sporadic reading of National Guidelines 🡪 unfamiliar with its content - National Guidelines are perceived as relevant and useful   **Interaction with colleagues**   - Learning from collaboration with CHC colleagues - Support from higher level of the healthcare system   **Other channels**   - Learning from mass media but not from the internet - Learning from books - Gain knowledge from drug companies   - Learning by doing   **Level of knowledge and skills**   - Staff level of knowledge and skills is low - Broad repertoire needed for working at a CHC   **Integration of knowledge and practices**   - Lack of awareness and knowledge of evidence-based practices - Evidence-based practices are known but not implemented and used - Lack of patient awareness or resources - Introduction of evidence-based practices | **Professional beliefs and use**   - CHC staff accepts TM procedures in the communities - Staff at CHC advise and provide instructions for caretakers about TM - Staff at CHC believes in and uses TM - Chinese TM is problematic   **Presence in general population**   - Knowledge about TM passes on from generations and within communities - Belief in TM is no longer strong - Availability and belief of TM is a geographic issue - Traditional beliefs about delivery, postpartum period, and death | **Healthcare structures**   - Changes at CHC need direction and support from higher levels - Lack of feedback from CHC to higher levels - Higher levels blame CHCs for improper care and referral   **Geographic location**   - Poor roads 🡪 poor quality of care program - Difficult to attend basic training because of long distances and family situations - Lack of skilled staff in rural locations   **Number of patients**   - - Patients bypass CHCs 🡪 low workload 🡪 degenerating knowledge/skills   **Data management/reporting**   - Data management and reporting important but fails to function efficiently   **Availability of material resources**   - Lack of necessary equipment - Unused resources - Drugs not available at the community level   **Commitment**   - Time and commitment are required for change of knowledge and practice to take place - Commitment needed for learning - Village health workers are important but low paid and not always committed |

## ^1^ National standards and guidelines for reproductive healthcare services (2003) by the Ministry of Health in Vietnam
